# Voluntary Exercise Decreases Atherosclerosis in Nephrectomised ApoE Knockout Mice

**DOI:** 10.1371/journal.pone.0120287

**Published:** 2015-03-23

**Authors:** Cecilia M. Shing, Robert G. Fassett, Jonathan M. Peake, Jeff S. Coombes

**Affiliations:** 1 School of Health Sciences, University of Tasmania, Launceston, Tasmania, Australia; 2 School of Human Movement Studies, University of Queensland, Brisbane, Queensland, Australia; 3 School of Biomedical Sciences, Queensland University of Technology, Brisbane, Queensland, Australia; The Ohio State University, UNITED STATES

## Abstract

Cardiovascular disease is the main cause of morbidity and mortality in patients with kidney disease. The effectiveness of exercise for cardiovascular disease that is accelerated by the presence of chronic kidney disease remains unknown. The present study utilized apolipoprotein E knockout mice with 5/6 nephrectomy as a model of combined kidney disease and cardiovascular disease to investigate the effect of exercise on aortic plaque formation, vascular function and systemic inflammation. Animals were randomly assigned to nephrectomy or control and then to either voluntary wheel running exercise or sedentary. Following 12-weeks, aortic plaque area was significantly (p<0.05, *d*=1.2) lower in exercising nephrectomised mice compared to sedentary nephrectomised mice. There was a strong, negative correlation between average distance run each week and plaque area in nephrectomised and control mice (r=–0.76, p=0.048 and r=–0.73, p=0.062; respectively). *In vitro* aortic contraction and endothelial-independent and endothelial-dependent relaxation were not influenced by exercise (p>0.05). Nephrectomy increased IL-6 and TNF-α concentrations compared with control mice (p<0.001 and p<0.05, respectively), while levels of IL-10, MCP-1 and MIP-1α were not significantly influenced by nephrectomy or voluntary exercise (p>0.05). Exercise was an effective non-pharmacologic approach to slow cardiovascular disease in the presence of kidney disease in the apolipoprotein E knockout mouse.

## Introduction

The progression of chronic kidney disease (CKD) is associated with increased cardiovascular morbidity and mortality [1], that is greater than traditional risk factors such dyslipidemia, hypertension and diabetes alone can account for. In an attempt to understand potential mechanisms for this increased mortality, significant attention has been given to the interaction between the kidney and heart [2]. Strategies targeted at reducing non-traditional risk factors such as oxidative stress, inflammation and endothelial dysfunction may prove effective treatment in the early stages of CKD, with the potential to slow the decline in kidney function and associated cardiovascular disease [3].

Exercise has proven beneficial in reducing cardiovascular disease risk factors [4]. In apolipoprotein E knockout (apoE^-^/^-^) mice, a strain that shows accelerated development of atherosclerotic lesions when on a normal chow diet [5], exercise reduces atherosclerotic lesions [6] and enhances endothelial-dependent vasorelaxation [7]. While exercise is effective at influencing the severity and composition of atherosclerotic plaque in cardiovascular disease it is unknown if exercise is beneficial in combined CKD and cardiovascular disease where exercise may not ameliorate plaque as effectively. In apoE^-^/^-^ mice, which have a reduced glomerular filtration rate compared with wild type controls [8], the ensuing CKD accelerates atherosclerosis [9–11] and increases thickening of aortic valves [12]. Whereas the benefits of exercise for cardiovascular disease risk are well recognised, the effect of exercise on plaque formation in a model of cardiovascular disease and CKD is yet to be determined.

To date, treatments such as statins have been relatively ineffective for reducing the progression of cardiovascular disease morbidity and mortality in this population [13], indicating that the combination of these two diseases may be resistant to existing drug therapies. Exercise may prove an effective non-pharmacologic approach to prevent or slow cardiovascular disease in the presence of kidney disease, although there is no research to date that has investigated if exercise is effective at reducing plaque in the presence of CKD, where the composition of plaque is different from plaques in cardiovascular disease alone [14]. The aim of the present study was to determine the influence of exercise on the development of atherosclerosis, vascular dysfunction and systemic inflammation in apoE^-^/^-^ mice with 5/6 nephrectomies.

## Methods

### Animals and Diet

Ten-week-old male apoE^-^/^-^ mice (B6.129P2-Apoe ^tm1Unc^/Arc, Animal Resource Centre, Canning Vale, Western Australia) were randomly divided to undergo a 5/6 nephrectomy or sham operation (control) (surgical procedures are explained in the following paragraph). One week following surgery mice were then randomly allocated to one of the following groups: 1) control (Cont) (n = 10), 2) 5/6 nephrectomy (Nx) (n = 10), 3) control undertaking voluntary exercise (Cont + Ex) (n = 10) and 4) 5/6 nephrectomy undertaking voluntary exercise (Nx + Ex) (n = 10). Mice were housed in separate cages and maintained on a 12-hour light/dark photo-period with a room temperature of 21 ± 2°C. All mice were fed standard mouse chow and water *ad libitum*. The University of Tasmania Animal Ethics Committee in accordance with the Australian Code of Practice approved all procedures for the Care and Use of Animals for the Scientific Purposes (A0009788). All efforts were made to minimize suffering.

### Surgical Procedures

CKD was induced using a 5/6 nephrectomy procedure in which the right kidney was removed and the lower branch of the left renal artery was ligated to produce approximately two thirds area with renal ischemia. To ensure uniform kidney damage, thermal injury was induced by angled point cautery in the fully exteriorized left kidney cortex [15]. The infarcted left kidney was not removed. Sham operated mice (Cont) were anesthetized and subjected to the same surgical procedure except for subtotal nephrectomy. Induction of anaesthesia was with up to 5% isoflurane mixed with 100% oxygen and this was then maintained with 2% isoflurane and 100% oxygen, delivered using a precision vaporizer. Buprenorphine was given for analgesia after surgery at a dose of 0.05 mg/kg subcutaneously every 3–5 hours for up to 24 hours.

### Voluntary Wheel Exercise

Cont + Ex and Nx + Ex mice were housed in cages (46 × 26 × 14.5 cm) supplied with a running wheel (11.5 cm diameter). The running wheel was equipped with a tachometer (DIGI-X8, PRO, The Netherlands) to determine total running distance, average speed and total running time per 24-hour period. The mice ran, predominantly at night, for a total of 12 weeks. Non-exercising mice (Cont and Nx) were housed in cages without a running wheel. Voluntary running was chosen over forced exercise to ensure that the responses were reflective of normal physiological functioning in the mouse where diurnal patterns were maintained and exercise mode was not removed from normal mouse behaviour.

### Anaesthesia

After 12-weeks of voluntary exercise or no exercise, mice were exsanguinated following the administration of 90mg/kg Pentobarbital Sodium (Nembutal) via an intraperitoneal injection. Anaesthesia was monitored prior to opening the chest wall by testing for the presence of reflexes. Blood was collected by the insertion of a 25-gauge syringe into the vena cava. The heart was then removed and placed in 10% buffered formalin solution.

### Vascular function

Immediately after exsanguination, the thoracic aorta was removed and dissected into three 3-mm aortic rings. These rings were then suspended and hooked to a force transducer in a warmed organ bath (37 ± 0.5°C) containing gassed (95% O_2_ and 5% CO_2_) physiological solution (in mM: NaCl 136.9, KCl 5.4, MgCl_2_ 1.05, NaH_2_PO_4_ 0.42, NaHCO_3_ 22.6, CaCl_2_ 1.8, glucose 5.5, ascorbic acid 0.28 and Na_2_EDTA 0.05). Smooth muscle cell contractile function was determined by a cumulative concentration-response curve to increasing concentrations of noradrenaline (concentration 10^-9^ to 10^-5^ M). Endothelial control of vascular relaxation (endothelium-dependent) was determined by adding the muscarinic agonist acetylcholine (concentration 10^-9^ to 10^-5^ M) following a 70% submaximal pre-contraction to noradrenaline. We assessed smooth muscle cell vasorelaxant function (endothelium-independent relaxation) was assessed by adding sodium nitroprusside (concentration 10^-9^ to 10^-5^ M) following pre-contraction with noradrenaline. The change in isometric force was measured using Grass FT03 force transducers (Grass, MA, USA) connected to a PowerLab chart recording system using Chart 4.0 recording software (AD Instruments, Sydney, NSW, Australia).

### Systemic Inflammation

The plasma concentration of interleukin (IL)-6, IL-10, monocyte chemotactic protein (MCP)-1, macrophage inflammatory protein (MIP)-1α, KC and tumour necrosis factor (TNF)-α were assayed simultaneously using the multiplex assay technique in a suspension array system according to the manufacturer’s instructions (Mouse Cytokine/Chemokine LINCOplex KIT, LINCO Research Inc., St. Charles, MO, USA). The contribution of the nephrectomy to systemic cytokine concentrations was determined by comparing the sedentary nephrectomy and control groups. While voluntary wheel running in mice has been shown to be protective against inflammation with forced treadmill exercise exacerbating inflammation [16], the mice running wheels were not locked the day prior to surgery and as such the systemic cytokine levels are reflective of approximately six hours following the final nocturnal activity (as running-wheel activity aligns with the environmental light-dark cycle). The contribution of the nephrectomy to systemic cytokine concentrations following exercise was determined by a comparison to cytokine concentrations of the control exercise group.

### Plaque Formation

The heart, including the aortic root, was removed and fixed in 10% buffered formalin to measure the surface area covered by lipid-stained lesions. The aorta root was serially sectioned every 4 μm from the cardiac end until the aortic sinuses appeared. Ten μm thick sections were then taken every 20 μm spanning 300 μm of the aortic root from aortic leaflets and upward and the slides were stained for oil red O. Slides were photographed with an Aperio Scanscope XT histology slide scanner (Leica Biosystems, Pty Ltd, Melbourne, Australia) and plaque area was determined using Analytical Image Software (Ontario, Canada). Aortic root plaque area was expressed as the volume of plaque over 300 μm.

### Plasma Creatinine

Plasma creatinine was determined on non-haemolysed samples using a colorimetric picric acid method previously described [17] and reported to have a correlation of r^2^ = 0.96 with a HPLC method. The assay was performed on a Cobas Mira (Roche Diagnostics, NSW, Australia).

### Statistical Analyses

Data were first tested for normality and non-normally distributed data were log transformed prior to analysis. Main effects of group were determined by an ANOVA (group x time) with Bonferroni post hoc testing. Significance was set at P < 0.05. Data were analysed using Prism 5 for Windows (GraphPad Software, Inc., CA, USA). For systemic markers and plaque area Cohen’s *d* was calculated between groups (difference in group means, divided by pooled standard deviation) and the effect size was interpreted as small = 0.2, moderate = 0.5 and large = 0.8.[18] Data are presented as mean ± SD unless otherwise indicated.

## Results

### Distance Run

**Fig 1:**
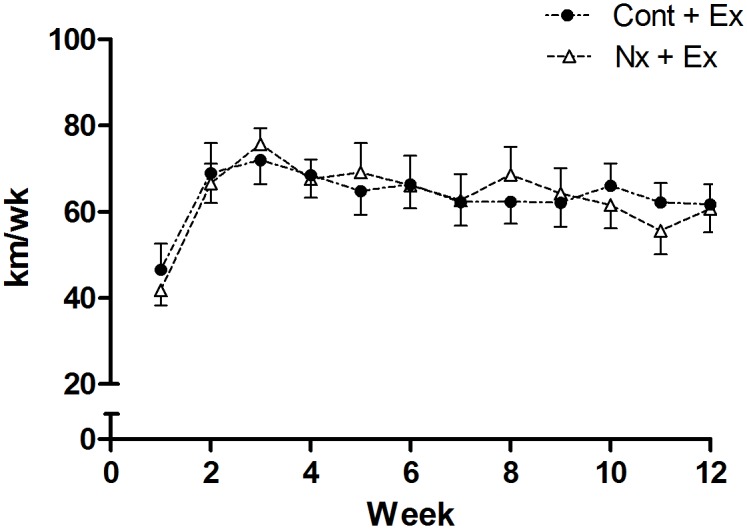
Average weekly voluntary running distance in control (Cont + Ex) and nephrectomy (Nx + Ex) mice.

Total running distance over 12-weeks was not significantly different between Cont + Ex (905 ± 309 km) and Nx + Ex groups (826 ± 198 km) (p = 0.39) with an average weekly distance over the 12 weeks of 63.7 ± 6.3 km and 63.3 ± 8.5 km, respectively (p = 0.92) (Fig. 1). Average speed was similar between the Cont + Ex (0.46 ± 0.05m·s^-1^) and Nx + Ex groups (0.47 ± 0.15 m·s^-1^) (p = 0.70).

### Body Weight

At 12-weeks, control mice (30.1 ± 3.1 g) were significantly heavier than Nx + Ex mice (26.8 ± 1.5 g) (p = 0.013) and sedentary Nx mice (27.4 ± 1.7 g) (p = 0.046). Exercising control mice (28.2 ± 2.2 g) were not significantly different to other groups (p >0.05)

### Creatinine

Plasma creatinine concentration was significantly higher in Nx mice (217.9 ± 9 μmol/L) compared with control mice (82.7 ± 69.1 µmol/L) (p = 0.006, *d* = 1.65), and also in Nx + ex mice (Nx + Ex = 290.5 ± 50.3 µmol/L) compared with control + Ex (131.6 ± 109.0 µmol/L) (p = 0.003, *d* = 2.0). There was a large increase (*d* = 1.0) in plasma creatinine concentration of exercising NX mice compared to sedentary Nx mice (p = 0.08), and a moderate increase in control exercising mice compared to sedentary mice (p = 0.3, *d* = 0.55).

### Total Plaque Area

**Fig 2:**
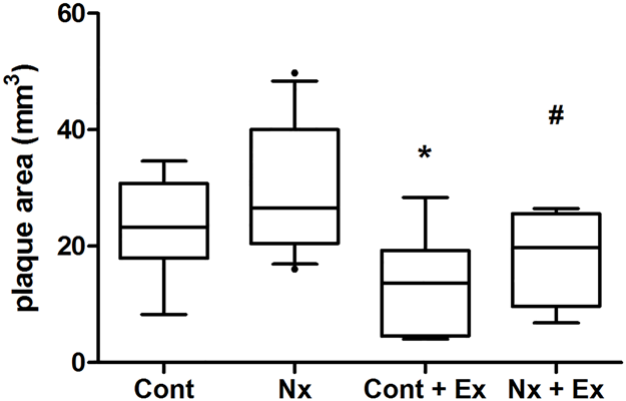
Total plaque area. Nx + Ex: nephrectomy and exercise, Nx: sedentary nephrectomy, Cont + Ex = sham exercise mice, Cont: sham sedentary mice. Lines represents the 25^th^ percentile, median and 75^th^ percentile while whiskers represent 10–90^th^ percentile range. *sig diff from Cont, # = sig diff from Nx.

**Fig 3:**
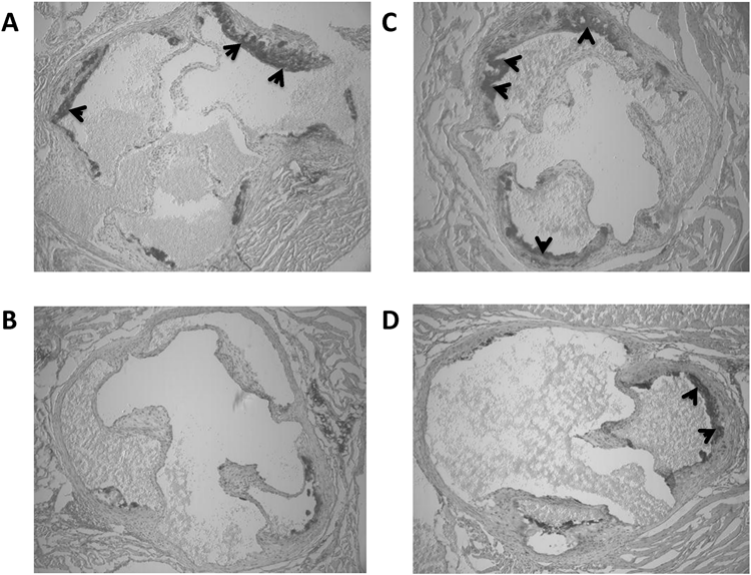
Representative images of aortic sinus in control and Nx apoE-/- mice undertaking voluntary exercise stained with Oil Red O (depicted as darker areas and indicated with arrows). A = control, B = control + exercise, C = nephrectomy, and D = nephrectomy + voluntary exercise.

Plaque area was significantly different between exercising and sedentary Nx and control groups (p = 0.007) (Fig. 2). Exercise was associated with a large reduction in plaque area in Nx mice (18.11 ± 7.75 mm^3^, p = 0.024, *d* = 1.18) and control mice (13.25 ± 9.21 mm^3^, p = 0.045, *d* = 1.14) when compared with sedentary Nx (29.22 ± 10.99 mm^3^) and control mice (23.37 ± 8.44 mm^3^), respectively (Fig. 3). There was a moderate increase in plaque area with Nx when compared to control mice (p = 0.20, *d* = 0.60). There was a significant, negative correlation between plaque area and average running distance per week in Nx mice (r = –0.76, p = 0.048), while there was also a trend for a strong, negative relationship between distance run and plaque area in control mice (r = –0.73, p = 0.062).

### Vascular Function

**Fig 4:**
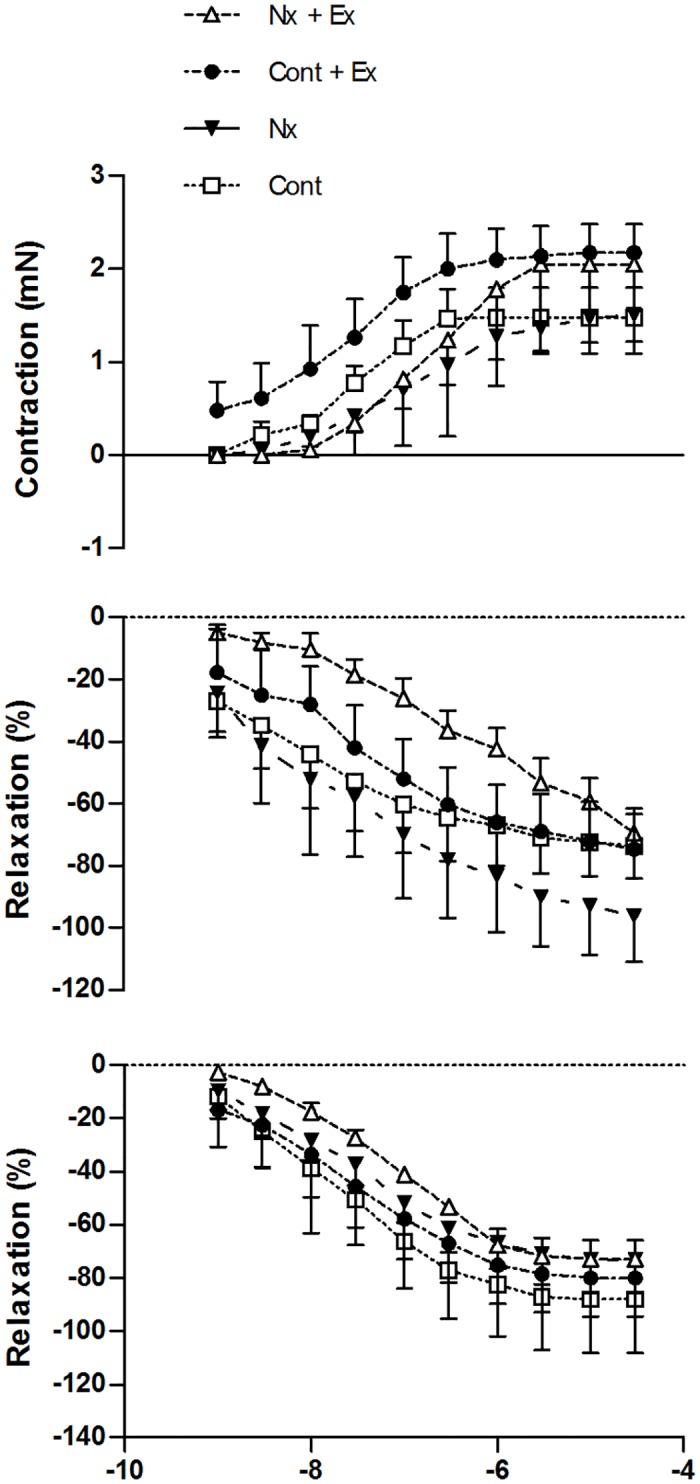
A. Aortic smooth muscle contraction (NA) dose response curve. B. Endothelial-dependent relaxation (Ach) dose response curve. C. Endothelial-independent (SNP) relaxation dose response curve. Vascular relaxation data expressed as % change in steady state tension following 70% submaximal pre-contraction with noradrenaline. Cont = control, Nx = sedentary nephrectomy, Cont + Ex = control and voluntary exercise, Nx + Ex = nephrectomy and voluntary exercise. Data are mean ± SE.

There was a significant effect of drug concentration but no main interaction of group x drug concentration for noradrenaline-induced aortic vasoconstriction (p = 0.399), endothelial-dependent (p = 0.986) and endothelial-independent (p = 0.884) vascular relaxation (Fig. 4, Table 1).

**Table 1:** Maximal aortic contraction or relaxation, logEC50 and area under the curve (AUC) following 12 weeks of voluntary wheel running or no voluntary wheel running in nephrectomy and control apoE-/- mice.

	NA			SNP			Ach		
Group	Emax	LogEC50	AUC	Emin	LogIC50	AUC	Emin	LogIC50	AUC
**Cont**	1.50 (1.24 to 1.75)	-7.53 (-8.05 to-7.00)	4.55	-3.05 (-3.95 to-2.15)	-7.51 (-8.84 to-6.18)	9.24	-3.56 (-9.10 to 1.97)	-10.68 (very wide)	11.29
**Nx**	1.54 (1.14 to 1.94)	-6.96 (-7.59 to-6.32)	3.58	-1.69 (-2.08 to-1.31)	-7.26 (-7.99 to-6.52)	4.47	-2.19 (-4.67 to 0.29)	-6.80 (-9.32 to-4.27)	5.35
**Cont + Ex**	2.18 (1.76 to 2.60)	-7.53 (-8.39 to 6.67)	7.12	-2.43 (-3.35 to-1.50)	-7.55 (-9.55 to-5.59)	7.28	-2.19 (-3.22 to-1.16)	-7.51 (-10.08 to-4.95)	6.54
**Nx + Ex**	2.11 (1.12 to 3.10)	-6.77 (-7.78 to-5.75)	4.66	-3.61(-5.08 to-2.14)	-7.78 (-10.58 to-4.99)	11.53	-2.54 (-5.27 to 0.18)	-8.00 (-17.56 to 1.55)	7.23

Data are presented as mean (95% confidence interval). NA = noradrenaline, SNP = sodium nitroprusside, Ach = acetylcholine. Cont = control, Nx = sedentary nephrectomy, Cont + Ex = control and voluntary exercise, Nx + Ex = nephrectomy and voluntary exercise.

### Cytokines

**Fig 5:**
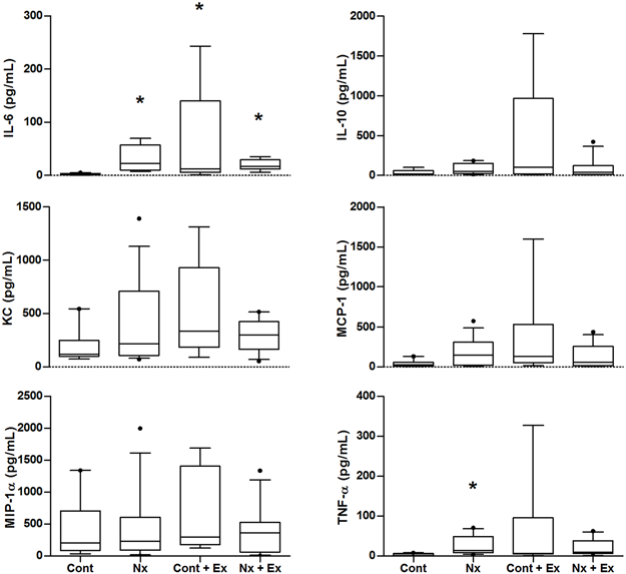
Plasma cytokine concentrations following 12 weeks of voluntary wheel running or no voluntary wheel running in nephrectomy and control apoE-/- mice. Box and whisker plots show the median with box extending from the 25^th^ to 75^th^ percentiles and whiskers representing the 10^th^ to 90^th^ percentiles. * = significantly different from Cont. Cont = control, Nx = sedentary nephrectomy, Cont + Ex = control and voluntary exercise, Nx + Ex = nephrectomy and voluntary exercise.

Plasma IL-6 concentration was significantly different between Nx, control and exercising mice (p<0.0001) (Fig. 5). Nx alone (*d* = 1.26) and exercise elevated IL-6 when compared to control (p<0.001) (Cont Ex p<0.001, *d* = 1.41; Nx + Ex p<0.001, *d* = 3.66). Exercise was associated with a large reduction in IL-6 levels in Nx mice when compared to Nx alone (*d* = 2.86) but this was not significant (p = 0.26). TNF-α was significantly different across groups (p = 0.043) with Nx elevating levels compared to control (p<0.05, d = 1.22). While there were no significant main effects of exercise or nephrectomy on IL-10 (p = 0.126), KC (p = 0.202), MCP-1 (p = 0.079) or MIP-1α (p = 0.574), exercise was associated with very large reductions in KC (*d* = 2.50), MCP-1 (*d* = 1.95) or MIP-1α (*d* = 1.74) and a very large increase in IL-10 (*d* = 1.78), which has anti-inflammatory properties, in Nx mice compared to sedentary Nx mice. While not statistically significant, post-exercise cytokine concentrations were increased in control exercising mice compared to Nx exercising mice (*d* = 1.22 to 2.7).

## Discussion

The present study suggests that voluntary exercise is effective in limiting aortic plaque area in the apoE^-^/^-^ mouse model of CKD using 5/6 nephrectomy. Previous studies have shown that exercise reduces atherosclerosis in apoE^-^/^-^ mice while the effects of exercise on plaque formation in the presence of CKD have remained unknown.

The onset of CKD in apoE^-^/^-^ mice accelerates atherosclerosis [9–11] and increases thickening of the aortic valves [12], in combination with elevating inflammatory cytokines. These mice also display reduced glomerular filtration rate and increased plasma creatinine levels compared with wild type controls [8], supporting that CKD is associated with the progression of atherosclerosis. In the present study, in comparison to control mice, the induction of CKD via nephrectomy was associated with a 25% increase in total plaque area, elevated circulating levels of inflammatory cytokines IL-6 and TNF-α, a reduction in body weight and an increase in creatinine concentration.

In support of previous findings [19–21], the current findings show that voluntary exercise was associated with significantly smaller total plaque area, 22% less than control apoE^-^/^-^ mice. The relationship between exercise volume and plaque severity is supported by the strong negative correlation between distance run and plaque area in both control and nephrectomised mice that exercised. Previous studies have reported an inverse relationship between average voluntary running distance per week and lesion area in the thoracoabdominal aorta over a period of 10-weeks in apoE^-^/^-^ mice concurrently consuming a high fat diet [22]. In contrast, Ajijola and colleagues reported that eight-weeks of voluntary exercise did not reduce atherosclerotic plaque area in the abdominal aorta or aortic sinus in apoE^-^/^-^ mice fed a high fat diet for 12-weeks prior to commencing exercise. The effectiveness of exercise to reduce plaque area may depend on the severity of atherosclerosis when exercise commences. Indeed, when Aijola et al repeated their experiment with apoE^-^/^-^ mice on a normal chow diet, voluntary exercise reduced aortic root lesion area [23]. Our findings support previous studies showing that exercise is effective at reducing atherosclerosis in the aortic sinus. Exercise results in a reduction in macrophage content and increase in smooth muscle cell count [20]. CKD in apoE^-^/^-^ mice does not increase macrophage infiltration in plaques, but does increase collagen content [24]. Whereas increased collagen content is associated with plaque stability, inflammation and elevated lipid content are also important contributors to plaque instability [25]. This is the first investigation to show that exercise is effective at reducing atherosclerosis burden in apoE^-^/^-^ mice with kidney disease. In the present study, lesion area was smaller, but as we did not measure stability it remains unknown if exercise improves plaque stability in combined kidney disease and atherosclerosis.

In cardiovascular disease alone hypertension has been related to atherosclerosis development however, blood pressure in the apoE^-^/^-^ mouse is unaffected by CKD [11, 15] and as such unlikely to contribute to vascular dysfunction. The increased burden of cardiovascular disease in the presence of CKD has been attributed to disturbances in calcium and phosphate [26], inflammation [27] and oxidative stress [28]. These factors impair vascular function, which often precedes formation of atherosclerosis. Functional or morphological changes to the endothelium stimulate atherogenesis, and evidence suggests that endothelial cell dysfunction is the first step in this process. Although 5/6 nephrectomy increased the aortic sinus plaque burden in the present study, it did not alter maximal contraction or relaxation of the thoracic aorta. Previous studies have reported endothelial dysfunction in uremic apoE^-^/^-^ mice fed a Western diet [22]. In contrast, endothelial dysfunction in plaque free aorta segments remains unchanged in non-uremic apoE^-^/^-^ mice fed standard chow [29, 30]. Our findings suggest that vascular function of the thoracic aorta does not appear to be further impaired in apoE^-^/^-^ mice 12-weeks following kidney disease when mice consume a standard chow diet.

In the present study, nephrectomy alone raised the concentrations of IL-10, KC, MCP-1 and MIP-1α (*d* = 1.10 to 1.32) and significantly elevated IL-6 and TNF-α. However, the lack of any difference in vascular function between the nephrectomy and control groups suggests that these cytokines did not influence vascular function. In apoE^-^/^-^ mice without kidney disease on a normal chow diet (early atherosclerosis) exercise does not affect systemic cytokine concentrations [23]. However, exercise does attenuate the rise in systemic IL-1β, TNF, MCP-1α and IL-1α associated with advanced lesions resulting from high fat feeding [23]. Despite an increase in systemic IL-6 and TNF-α following nephrectomy, impairment of vascular function was not evident following a normal chow diet in the present study. Impairment of endothelial dependent relaxation (Fukao et al, 2010), elevated resting pro-inflammatory cytokine concentrations (Ajijola et al, 2009) and the progression of atherosclerosis in the apoE^-^/^-^ mouse are highly dependent on dietary fat content. Whether exercise is effective in reducing vascular impairments (induced by high fat feeding) in the presence of kidney disease, where systemic cytokine concentrations are elevated up to 10 fold, remains to be determined.

While exercise is associated with an acute increase in both pro- and anti-inflammatory cytokines [31], long term exercise training has been shown to reduce resting systemic pro-inflammatory cytokines in patients with cardiovascular disease [32] and mice with advanced, but not early, atherosclerotic lesions [23]. In the present study post-exercise (6 hours following the last exercise bout) cytokine concentrations were determined. Interestingly, there was a large to very large increase in post-exercise cytokine concentrations in control mice compared to nephrectomy mice (*d* = 1.22 to 2.7). Exercise induced increase in cytokines, particularly IL-6 and IL-10, is often dependent on exercise intensity [33, 34]. While both nephrectomy and control groups covered similar distances it is possible that the intensity of the exercise was reduced in the nephrectomy mice. Exercise capacity is reduced in end stage kidney disease [35] and there is evidence that left ventricular hypertrophy, which has been shown to reduce exercise capacity, is present in early to moderate stage CKD [36]. As mice were exercising voluntarily it is still of note that the volume of exercise the nephrectomy mice were able to engage in conferred a benefit to reduce atherosclerotic plaque area.

There is a pathophysiological interaction between the kidney and heart which is associated with a number of systemic disturbances such as elevated levels of uremic toxins and inflammation that adversely affect the cardiovascular system [2]. Although we showed that voluntary aerobic exercise results is less aortic plaque, plasma creatinine concentration did not change in response to exercise. This finding may suggest that kidney dysfunction in the presence of cardiovascular disease was not influenced by voluntary aerobic exercise. A recent study by Boor et al [37] investigated the effects of exercise in a rat model of diabetic nephropathy. They discovered that exercise reduced tubulointerstitial fibrosis, despite plasma creatinine concentrations remaining unchanged, perhaps due to changes in muscle mass. Creatinine concentrations in the present study were reflective of moderate to severe CKD at 12 weeks [38, 39]. The effects of exercise on CKD progression in the presence of cardiovascular disease therefore warrants further investigation.

In the ApoE-/- mouse model with 5/6 nephrectomy voluntary exercise for 12-weeks was effective in reducing plaque area. The progression of CKD is associated with increased cardiovascular morbidity and mortality [1], and as such reducing plaque formation may influence CKD mortality. Future work should determine the effect of exercise on both CKD and cardiovascular disease progression with a view to determining the mechanisms behind less plaque formation in CKD associated cardiovascular disease, particularly as exercise modifies numerous physiological variables including blood pressure and adiposity. Voluntary aerobic exercise is an effective means of reducing atherosclerosis both in the presence of, and without kidney disease in this model, and may be an effective therapy to reduce plaque formation in early stage CKD

## References

[1] Levin A. Clinical epidemiology of cardiovascular disease in chronic kidney disease prior to dialysis. Semin Dial. 2003;16: 101–105. 12641872 10.1046/j.1525-139x.2003.16025.x

[2] Ronco C, Chionh CY, Haapio M, Anavekar NS, House A, Bellomo R. The cardiorenal syndrome. Blood Purif. 2009;27: 114–126. 10.1159/000167018 19169027

[3] Tonelli M, Sacks F, Pfeffer M, Jhangri GS, Curhan G. Biomarkers of inflammation and progression of chronic kidney disease. Kidney Int. 2005;68: 237–245. 15954913 10.1111/j.1523-1755.2005.00398.x

[4] Hilberg T. Physical activity in the prevention of cardiovascular diseases. Epidemiology and mechanisms. Hamostaseologie. 2008;28: 9–15. 18278156

[5] Plump AS, Smith JD, Hayek T, Aalto-Setala K, Walsh A, Verstuyft JG, et al Severe hypercholesterolemia and atherosclerosis in apolipoprotein E-deficient mice created by homologous recombination in ES cells. Cell. 1992;71: 343–353. 1423598 10.1016/0092-8674(92)90362-g

[6] Okabe TA, Shimada K, Hattori M, Murayama T, Yokode M, Kita T, et al Swimming reduces the severity of atherosclerosis in apolipoprotein E deficient mice by antioxidant effects. Cardiovasc Res. 2007;74: 537–545. 17374527 10.1016/j.cardiores.2007.02.019

[7] Laufs U, Wassmann S, Czech T, Munzel T, Eisenhauer M, Bohm M, et al Physical inactivity increases oxidative stress, endothelial dysfunction, and atherosclerosis. Arterioscler Thromb Vasc Biol. 2005;25: 809–814. 15692095 10.1161/01.ATV.0000158311.24443.af

[8] Balarini CM, Oliveira MZ, Pereira TM, Silva NF, Vasquez EC, Meyrelles SS, et al Hypercholesterolemia promotes early renal dysfunction in apolipoprotein E-deficient mice. Lipids Health Dis. 2011;10: 220 10.1186/1476-511X-10-220 22117541 PMC3247872

[9] Bro S, Bentzon JF, Falk E, Andersen CB, Olgaard K, Nielsen LB. Chronic renal failure accelerates atherogenesis in apolipoprotein E-deficient mice. J Am Soc Nephrol. 2003;14: 2466–2474. 14514724 10.1097/01.asn.0000088024.72216.2e

[10] Bro S, Binder CJ, Witztum JL, Olgaard K, Nielsen LB. Inhibition of the renin-angiotensin system abolishes the proatherogenic effect of uremia in apolipoprotein E-deficient mice. Arterioscler Thromb Vasc Biol. 2007;27: 1080–1086. 17347482 10.1161/ATVBAHA.107.139634

[11] Buzello M, Tornig J, Faulhaber J, Ehmke H, Ritz E, Amann K. The apolipoprotein e knockout mouse: a model documenting accelerated atherogenesis in uremia. J Am Soc Nephrol. 2003;14: 311–316. 12538731 10.1097/01.asn.0000045048.71975.fc

[12] Simolin MA, Pedersen TX, Bro S, Mayranpaa MI, Helske S, Nielsen LB, et al ACE inhibition attenuates uremia-induced aortic valve thickening in a novel mouse model. BMC Cardiovasc Disord. 2009;9: 10 10.1186/1471-2261-9-10 19257900 PMC2663538

[13] Caravaca F, Alvarado R, Garcia-Pino G, Martinez-Gallardo R, Luna E. During the pre-dialysis stage of chronic kidney disease, which treatment is associated with better survival in dialysis? Nefrologia. 2014;34: 469–476. 10.3265/Nefrologia.pre2014.Apr.12277 25036060

[14] Pelisek J, Assadian A, Sarkar O, Eckstein HH, Frank H. Carotid plaque composition in chronic kidney disease: a retrospective analysis of patients undergoing carotid endarterectomy. Eur J Vasc Endovasc Surg. 2010;39: 11–16. 10.1016/j.ejvs.2009.09.024 19906548

[15] Gagnon RF, Duguid WP. A reproducible model for chronic renal failure in the mouse. Urol Res. 1983;11: 11–14. 6857877 10.1007/BF00272702

[16] Cook MD, Martin SA, Williams C, Whitlock K, Wallig MA, Pence BD, et al Forced treadmill exercise training exacerbates inflammation and causes mortality while voluntary wheel training is protective in a mouse model of colitis. Brain Behav Immun. 2013;33: 46–56. 10.1016/j.bbi.2013.05.005 23707215 PMC3775960

[17] Keppler A, Gretz N, Schmidt R, Kloetzer HM, Groene HJ, Lelongt B, et al Plasma creatinine determination in mice and rats: an enzymatic method compares favorably with a high-performance liquid chromatography assay. Kidney Int. 2007;71: 74–78. 17082757 10.1038/sj.ki.5001988

[18] Cohen J. A power primer. Psychological Bulletin. 1992;112: 155–159. 19565683 10.1037//0033-2909.112.1.155

[19] Kadoglou NP, Kostomitsopoulos N, Kapelouzou A, Moustardas P, Katsimpoulas M, Giagini A, et al Effects of exercise training on the severity and composition of atherosclerotic plaque in apoE-deficient mice. J Vasc Res. 2011;48: 347–356. 10.1159/000321174 21389732

[20] Pellegrin M, Miguet-Alfonsi C, Bouzourene K, Aubert JF, Deckert V, Berthelot A, et al Long-Term Exercise Stabilizes Atherosclerotic Plaque in ApoE Knockout Mice. Med Sci Sports Exerc. 2009;41: 2128–2135. 10.1249/MSS.0b013e3181a8d530 19915507

[21] Shon SM, Park JH, Nahrendorf M, Schellingerhout D, Kim JY, Kang BT, et al Exercise attenuates matrix metalloproteinase activity in preexisting atherosclerotic plaque. Atherosclerosis. 2011;216: 67–73. 10.1016/j.atherosclerosis.2011.01.036 21334624

[22] Fukao K, Shimada K, Naito H, Sumiyoshi K, Inoue N, Iesaki T, et al Voluntary exercise ameliorates the progression of atherosclerotic lesion formation via anti-inflammatory effects in apolipoprotein E-deficient mice. J Atheroscler Thromb. 2010;17: 1226–1236. 20808053 10.5551/jat.4788

[23] Ajijola OA, Dong C, Herderick EE, Ma Q, Goldschmidt-Clermont PJ, Yan Z. Voluntary running suppresses proinflammatory cytokines and bone marrow endothelial progenitor cell levels in apolipoprotein-E-deficient mice. Antioxid Redox Signal. 2009;11: 15–23. 10.1089/ars.2008.2092 18837653 PMC2933158

[24] Massy ZA, Ivanovski O, Nguyen-Khoa T, Angulo J, Szumilak D, Mothu N, et al Uremia accelerates both atherosclerosis and arterial calcification in apolipoprotein E knockout mice. J Am Soc Nephrol. 2005;16: 109–116. 15563564 10.1681/ASN.2004060495

[25] Shah PK. Role of inflammation and metalloproteinases in plaque disruption and thrombosis. Vasc Med. 1998;3: 199–206. 9892512 10.1177/1358836X9800300304

[26] Torres PA, De Broe M. Calcium-sensing receptor, calcimimetics, and cardiovascular calcifications in chronic kidney disease. Kidney Int. 2012;82: 19–25. 10.1038/ki.2012.69 22437409

[27] Rosner MH, Ronco C, Okusa MD. The role of inflammation in the cardio-renal syndrome: a focus on cytokines and inflammatory mediators. Semin Nephrol. 2012;32: 70–78. 10.1016/j.semnephrol.2011.11.010 22365165

[28] Wilcox CS. Asymmetric dimethylarginine and reactive oxygen species: unwelcome twin visitors to the cardiovascular and kidney disease tables. Hypertension. 2012;59: 375–381. 10.1161/HYPERTENSIONAHA.111.187310 22215715 PMC3266466

[29] Crauwels HM, Van Hove CE, Holvoet P, Herman AG, Bult H. Plaque-associated endothelial dysfunction in apolipoprotein E-deficient mice on a regular diet. Effect of human apolipoprotein AI. Cardiovasc Res. 2003;59: 189–199. 12829190 10.1016/s0008-6363(03)00353-5

[30] Meyrelles SS, Peotta VA, Pereira TM, Vasquez EC. Endothelial dysfunction in the apolipoprotein E-deficient mouse: insights into the influence of diet, gender and aging. Lipids Health Dis. 2011;10: 211 10.1186/1476-511X-10-211 22082357 PMC3247089

[31] Pedersen BK. Special feature for the Olympics: effects of exercise on the immune system: exercise and cytokines. Immunol Cell Biol. 2000;78: 532–535. 11050536 10.1111/j.1440-1711.2000.t01-11-.x

[32] Kim YJ, Shin YO, Bae JS, Lee JB, Ham JH, Son YJ, et al Beneficial effects of cardiac rehabilitation and exercise after percutaneous coronary intervention on hsCRP and inflammatory cytokines in CAD patients. Pflugers Arch. 2008;455: 1081–1088. 17906875 10.1007/s00424-007-0356-6

[33] Fischer CP. Interleukin-6 in acute exercise and training: what is the biological relevance? Exerc Immunol Rev. 2006;12: 6–33. 17201070

[34] Peake JM, Suzuki K, Hordern M, Wilson G, Nosaka K, Coombes JS. Plasma cytokine changes in relation to exercise intensity and muscle damage. Eur J Appl Physiol. 2005;95: 514–521. 16151834 10.1007/s00421-005-0035-2

[35] Zuo ML, Yue WS, Yip T, Ng F, Lam KF, Yiu KH, et al Prevalence of and associations with reduced exercise capacity in peritoneal dialysis patients. Am J Kidney Dis. 2013;62: 939–946. 10.1053/j.ajkd.2013.05.016 23886613

[36] Rugale C, Du Cailar G, Fesler P, Ribstein J, Mourad G, Mimran A. Effect of early stage kidney disease on cardiac mass: comparison to post-donation renal function. Am J Nephrol. 2013;38: 168–173. 10.1159/000353931 23941801

[37] Boor P, Celec P, Behuliak M, Grancic P, Kebis A, Kukan M, et al Regular moderate exercise reduces advanced glycation and ameliorates early diabetic nephropathy in obese Zucker rats. Metabolism. 2009;58: 1669–1677. 10.1016/j.metabol.2009.05.025 19608208

[38] Hobo A, Yuzawa Y, Kosugi T, Kato N, Asai N, Sato W, et al The growth factor midkine regulates the renin-angiotensin system in mice. J Clin Invest. 2009;119: 1616–1625. 10.1172/JCI37249 19451697 PMC2689110

[39] Komura N, Kihara S, Sonoda M, Maeda N, Tochino Y, Funahashi T, et al Increment and impairment of adiponectin in renal failure. Cardiovasc Res. 2010;86: 471–477. 10.1093/cvr/cvp415 20035033

